# T-5224, a selective inhibitor of c-Fos/activator protein-1, improves survival by inhibiting serum high mobility group box-1 in lethal lipopolysaccharide-induced acute kidney injury model

**DOI:** 10.1186/s40560-015-0115-2

**Published:** 2015-11-14

**Authors:** Mari Ishida, Masaaki Ueki, Jun Morishita, Masaki Ueno, Shunichi Shiozawa, Nobuhiro Maekawa

**Affiliations:** Department of Anesthesia, Hyogo Rehabilitation Centre Central Hospital, Kobe, Hyogo 651-2181 Japan; Division of Anesthesia, Nishiwaki Municipal Hospital, 652-1 Shimotoda, Nishiwaki, Hyogo 677-0043 Japan; Department of Emergency and Critical Care Medicine, Kobe University Hospital, Kobe, Hyogo 650-0017 Japan; Department of Anesthesia, Higashiosaka City General Hospital, Osaka, 578-8588 Japan; Department of Pathology and Host Defense, Faculty of Medicine, Kagawa University, Kagawa, 761-0793 Japan; Department of Internal Medicine, Kyushu University Beppu Hospital, Oita, 874-0838 Japan

**Keywords:** T-5224, C-Fos/activator protein-1, Lipopolysaccharide (LPS), Tumor necrosis factor-alpha (TNF-α), Interleukin-10 (IL-10), High mobility group box-1 (HMGB-1)

## Abstract

**Background:**

Sepsis is a potentially fatal syndrome mediated by an early [e.g., tumor necrosis factor-alpha (TNF-α)] and late [high mobility group box-1 (HMGB-1)] proinflammatory cytokine response to infection. Sepsis-induced acute kidney injury (AKI) is associated with a high mortality. C-Fos/activator protein-1 (AP-1) controls the transactivation of proinflammatory cytokines via AP-1 binding in the promoter region. T-5224 is a de novo small molecule inhibitor of c-Fos/AP-1 that controls gene expression of multiple proinflammatory cytokines. We investigated whether T-5224, a selective inhibitor of c-Fos/AP-1, improves survival in lethal lipopolysaccharide (LPS)-induced AKI by inhibiting early (TNF-α) and late (HMGB-1) proinflammatory cytokine response.

**Methods:**

Mice were divided into four groups (control, LPS, LPS + T-5224, and T-5224 only). Control mice were administered polyvinylpyrrolidone (PVP) solution orally, immediately after intraperitoneal (i.p.) saline injection. LPS mice were administered PVP solution orally immediately after i.p. LPS (10 mg/kg) injection. LPS + T-5224 mice were administered T-5224 orally (300 mg/kg) immediately after i.p. LPS injection. T-5224 mice were administered T-5224 orally (300 mg/kg) after i.p. saline injection. Serum concentrations of TNF-α, HMBG-1, and interleukin (IL)-10 were measured by enzyme-linked immunosorbent assay (ELISA). Serum blood urea nitrogen (BUN) and creatinine concentrations were commercially analyzed. Finally, histological examination was performed on the kidney.

**Results:**

Treatment with T-5224 decreased serum TNF-α and HMGB-1 levels and increased survival after LPS injection. Furthermore, T-5224 treatment decreased serum BUN and creatinine concentrations but increased serum IL-10 concentration. LPS-induced pathological changes in kidney were attenuated by T-5224 treatment.

**Conclusions:**

These results suggest that T-5224, a selective inhibitor of c-Fos/AP-1, inhibits expression of early and late proinflammatory cytokines, protecting mice from LPS-induced lethality. T-5224 is a potential approach for decreasing lethality in sepsis-induced AKI.

## Background

Sepsis is characterized by a severe systemic inflammatory response to infection. Sepsis causes multiple organ failure, and acute kidney injury (AKI) is a critical complication of sepsis [1]. Sepsis-induced AKI decreases survival prognosis and is associated with 70–80 % mortality in the intensive care unit [2]. This unacceptably high mortality rate has inspired researchers to better understand the molecular pathogenesis of AKI and seek improved therapeutic interventions.

Until recently, inflammatory reactions by cytokine cascades and oxidative stress were proposed as the molecular pathogenesis of sepsis-induced AKI [3]. High mobility group B-1 (HMGB-1) is a ubiquitous nuclear protein present in almost all eukaryotic cells, where it regulates transcription under normal conditions [4]. More importantly, unlike other proinflammatory cytokines such as tumor necrosis factor-alpha (TNF-α) and interleukin-1 beta (IL-1β), HMGB-1 was mplicated as a “late mediator” of lethal systemic inflammation in cytokine-mediated disease initiated by the Gram-negative bacterial product endotoxin [lipopolysaccharide (LPS)] [5]. Administration of a HMGB-1 neutralizing antibody, or of a drug that neutralizes HMGB-1, affords significant protection [5, 6]. These studies suggested that HMGB-1 may serve as a more effective therapeutic target for the treatment of lethal sepsis-induced AKI as opposed to other proinflammatory cytokines [7].

Activator protein-1 (AP-1) is a transcription factor composed of c-Fos and c-Jun proteins. C-Fos/AP-1 controls transactivation of multiple proinflammatory cytokines via promoter AP-1 binding motifs [8]. Tsuchida et al. designed and synthesized a selective inhibitor of c-Fos/AP-1 by three-dimensional (3D) pharmacophore modeling based on an X-ray crystal structure of the basic region-leucine zipper (bZIP) domain of the AP-1-DNA complex [9]. T-5224 is a de novo small molecule selective inhibitor of c-Fos/AP-1 that controls gene expression of multiple proinflammatory cytokines. Aikawa et al. demonstrated that oral administration of this selective inhibitor resolved collagen-induced arthritis in mice by inhibiting proinflammatory cytokines (e.g., IL-1 β, IL-6) [10]. Furthermore, we previously demonstrated that T-5224 prevented AKI by inhibiting serum early proinflammatory cytokines (TNF-α, IL-1β, IL-6) in a non-lethal model (LPS 6 mg/kg body weight) [11].

Based on selective inhibition of AP-1 signaling to control expression of proinflammatory cytokines, we sought to investigate whether T-5224 improved mortality by inhibiting early (TNF-α) and late (HMBG-1) responses in lethal LPS-induced AKI.

## Methods

### Animal experiments

Male 8-week-old C57BL/6 mice (20–25 g) were obtained from CLEA Japan, Inc. (Tokyo, Japan), housed in a specific pathogen-free grade environment, and provided food and water ad libitum under a 12-h light/dark cycle. All animal experiments were performed in accordance with the National Institutes of Health Guidelines under protocols approved by the Institutional Animal Care and Use Committee (permission number: P090416) of Kobe University (Kobe, Japan).

### Reagents and drugs

LPS (*Escherichia coli* serotype 0111:B4) was purchased from Sigma-Aldrich (St. Louis, MO, USA) and dissolved in normal saline for administration to mice. T-5224, which is 3-{5-[4-(cyclopentyloxy)-2-hydroxybenzoyl]-2-[(3-hydroxy-1,2-benzisoxazol-6-yl)methoxy]phenyl} propionic acid, was synthesized and generously donated by Toyama Chemical Co., Ltd. (Toyama, Japan). T-5224 was dissolved in polyvinylpyrrolidone (PVP) solution.

### Experimental design

Mice were randomly divided into four experimental groups: the control group (*n* = 58), administered PVP solution orally immediately after i.p. saline injection; the LPS group (*n* = 58), administered PVP solution orally immediately after i.p. LPS (10 mg/kg body weight) injection; the LPS + T-5224 group (*n* = 58), administered T-5224 (300 mg/kg body weight) immediately after LPS injection; and the T-5224 group (*n* = 58), administered T-5224 (300 mg/kg) orally, immediately after i.p. saline injection. In a series of preliminary studies (*n* = 7), we measured the TNF-α concentration at 1.5 h (2953.0 (2390.1–3659.9), pg/ml) and 3 h (742.6 (672.9–820.7), pg/ml) after LPS (10 mg/kg) injection. Furthermore, the effects of 30 mg/kg and 300 mg/kg of T-5224 on the serum TNF-α concentration 1.5 h after LPS injection were 2473.0 (2322.5–2658.9) pg/ml and 1702.0 (1610.6–1743.4) pg/ml.

Thus, we decided that the peak timing of serum TNF-α concentration was at 1.5 h after LPS injection and the optimal dose of T-5224 was 300 mg/kg.

### Measurement of serum TNF-α concentration

To measure the serum TNF-α concentration after LPS injection, 1 ml of blood was collected at 1.5 h after LPS injection via the femoral artery (*n* = 7 for each group). Blood was centrifuged (2500 × g, 10 min, 4 °C) and the serum collected and stored at −80 °C until analysis. Enzyme-linked immunosorbent assays (ELISAs) were carried out using a TNF-α ELISA kit (R&D systems, Minneapolis, MN, USA), according to the manufacturer’s protocols.

### Survival study and measurement of serum HMGB-1

To examine the protective effect of T-5224 on the survival rate after LPS injection, the survival rate was recorded every 6 h for 3 days in four groups (*n* = 20 for each group).

To measure serum HMGB-1 concentrations, 1 ml of blood was collected 18 h after LPS injection via the femoral artery (*n* = 7 for each group) [5]. ELISAs were carried out using a HMGB-1 ELISA kit (Shino-Test, Sagamihara, Kanagawa, Japan), according to the manufacturer’s protocols.

### Measurement of serum IL-10 serum and blood urea nitrogen (BUN) and creatinine concentrations

One milliliter of blood samples were collected at 1.5 h (serum IL-10, *n* = 7 for each group) or 18 h (serum BUN and creatinine, *n* = 7 for each group) after LPS injection via the femoral artery. Blood was centrifuged (2500 × g, 10 min, 4 °C) and the serum collected and stored at −80 °C until analysis. Serum IL-10 was measured using ELISA kits (Life Technologies, Carlsbad, CA, USA), according to the manufacturers’ protocols. The BUN and Cr concentrations were commercially analyzed (Special Reference Laboratories, Tokyo, Japan).

### Histological evaluation

Mice were sacrificed and kidneys were removed for histopathological observation at 24 h after LPS injection (*n* = 10 for each group). We evaluated the histology in 5 mice among 10 mice that we used for histological analysis study. A portion of the kidney was fixed immediately in 10 % formalin and embedded in wax according to a standard protocol. Samples were cut on a microtome into sections of 4 μm and stained with hematoxylin and eosin (H & E) to observe general cell morphology. Samples were examined with a light microscope.

### Statistical analyses

Statistical analyses were carried out using Kruskal-Wallis test for non-parametric data with Dunn’s multiple comparisons posttest. The survival curves compared using Kaplan-Meier methods followed by a log-rank test. Data are represented as medians with interquartile ranges. *P* < 0.05 was considered significant.

## Results

### Changes in serum TNF-α concentration

F-α is an inflammatory cytokine and plays an important role in LPS-induced early inflammatory responses. We examined the effect of T-5224 on serum TNF-α concentration at 1.5 h after LPS injection (Table 1). Serum TNF-α concentration in the LPS group (2738.0 (2429.7–3377.2) pg/ml) was higher than that of controls (39.5 (36.0–47.0) pg/ml, *P* < 0.001), and oral administration of T-5224 (300 mg/kg) immediately after LPS injection decreased serum TNF-α concentrations (1533.0 (1453.9–1602.6) pg/ml, *P* < 0.01, Table 1).

**Table 1:** Change in serum TNF-α, HMGB-1, IL-10, and BUN and creatinine concentrations

	Serum TNF-α (pg/ml, *n* = 7)	Serum HMGB-1 (ng/ml, *n* = 7)	Serum IL-10 (pg/ml, *n* = 7)	Serum BUN	Serum creatinine
				(mg/dl, *n* = 7)	
Control	39.5 (36.0–47.0)	19.8 (19.0–23.4)	59.5 (48.8–62.9)	29.6 (27.2–30.7)	0.11 (0.10–0.12)
LPS	2738.0 (2429.7–3377.2)**	169.8 (166.7–224.5)**	1361.0 (1256.3–1603.9)**	98.8 (95.3–102.8)**	0.35 (0.34–0.38)**
LPS + T-5224	1533.0 (1453.9–1602.6)**^,##^	132.8 (119.1–140.6)**^,##^	3165.0 (2897.8–3400.8)**^,##^	70.5 (68.4–76.8)**^,##^	0.28 (0.22–0.29)**^,##^
T-5224	32.0 (30.0–34.0)	19.4 (17.9–21.1)	64.9 (55.6–70.1)	28.0 (24.0–28.7)	0.10 (0.09–0.11)

Control: mice received PVP solution orally immediately after saline injection. LPS: mice received PVP solution orally immediately after LPS (10 mg/kg) injection. LPS + T-5224: mice received T-5224 (300 mg/kg) immediately after LPS injection. T-5224: mice received T-5224 (300 mg/kg) after saline injection. Data are represented as medians with interquartile ranges

*TNF-α* tumor necrosis factor-alpha, *HMGB-1* high mobility group box-1, *IL-10* interleukin-10, *BUN* blood urea nitrogen

**P < 0.01 compared with control group. ^##^ *P* < 0.01 compared with LPS group

### Changes of serum HMGB-1 concentration and survival

**Fig. 1:**
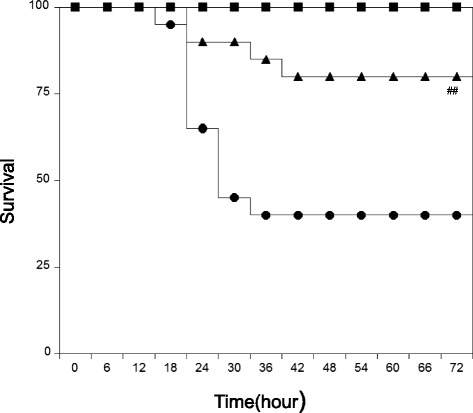
Effect of T-5524 on survival. T-5224 prevents LPS-induced lethality. The survival rate was recorded every 6 h for 3 days. *Open squares* are control mice. *Closed circles* are LPS mice. *Closed squares* are LPS + T-5224 mice. *Open circles* are T-5224 mice. *n* = 20 for each group. ^##^ *P* < 0.01 compared with LPS mice

HMGB-1 is a late mediator of lethal systemic inflammation in animal models of cytokine-mediated sepsis. Activated macrophages release HMGB-1 after a lag of 12–18 h [5]. Serum HMGB-1 concentration 18 h after LPS injection was significantly higher than that of controls (Table 1), and oral administration of T-5224 immediately after LPS injection inhibited serum HMGB-1 increases. Survival rates at 72 h post-LPS injection were 40 % (Fig. 1). However, in mice treated with T-5224, survival rates at the corresponding time point after LPS injection increased to 80 % (Fig. 1, *P* < 0.01).

### Changes in serum IL-10 concentration

We also examined serum concentrations of IL-10, an anti-inflammatory cytokine, 1.5 h after LPS injection [12], which were elevated in the LPS (1361.0 (1256.3–1603.9) pg/ml) relative to the control group (59.5 (48.8–62.9) pg/ml, *P* < 0.001). In addition, the concentration of IL-10 induced in LPS + T-5224 (3165.0 (2897.8–3400.8) pg/ml, *P* < 0.001, Table 1) increased compared with that of the LPS group.

### Changes in serum BUN and creatinine concentrations

We examined serum BUN and creatinine concentrations to evaluate kidney function. In the LPS group, serum BUN and creatinine concentrations were significantly increased at 18 h compared with those in the control group. The administration of T-5224 reduced serum BUN and creatinine concentrations at 18 h after LPS injection (Table 1).

### Histological changes in kidney tissue

**Fig. 2:**
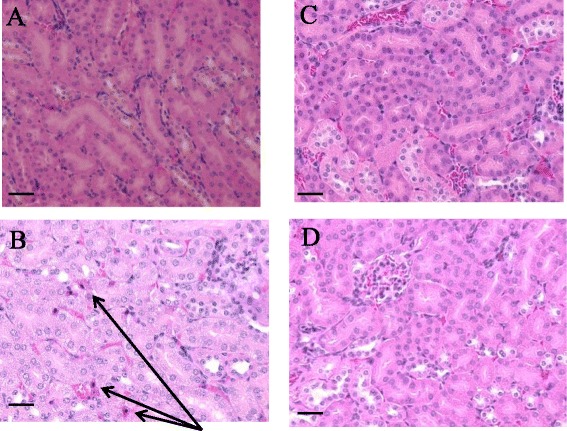
Effects of T-5224 on renal histopathology. T-5224 attenuated mild morphological damage, including necrotic degeneration with pyknotic nuclei. The sections shown were harvested 24 h after LPS injection and stained with H & E. **a** Control: mice received PVP solution orally immediately after i.p. saline injection. **b** LPS: LPS mice received PVP solution orally immediately after i.p. LPS (10 mg/kg body weight) injection. LPS caused necrotic degeneration with pyknotic nucleus (*arrow head*). **c** LPS + T-5224: LPS + T-5224 mice received T-5224 (300 mg/kg) immediately after LPS injection. There was less necrotic degeneration following T-5224 treatment. **d** T-5224: T-5224 mice received T-5224 (300 mg/kg) after i.p. saline injection. Scale bars = 100 μm. *n* = 5 for each group

To evaluate histopathological changes of the kidney, tissue sections were stained with H & E. Examination in the LPS group revealed mild morphological damage, including necrotic degeneration with pyknotic nuclei. In contrast, treatment with T-5224 reduced the extent of kidney injury (Fig. 2).

## Discussion

We investigated whether T-5224 improved mortality by inhibiting the early (TNF-α) and late (HMBG-1) proinflammatory response in lethal LPS-induced AKI, based on the idea that T-5524 inhibits c-Fos/AP-1, which transactivates proinflammatory cytokines via promoter AP-1 binding motifs [10]. Our results demonstrate that T-5224 blocks serum TNF-α, HMGB-1, BUN, and creatinine concentrations, reducing mortality of LPS-induced AKI. These findings suggest that T-5224 may be protective against lethal LPS-induced AKI.

It is well known that inflammation plays a key role in the pathogenesis and development of sepsis-induced AKI. There is an exaggerated stimulation of the normal host response to eradicate invasive pathogens, leading to excessive release of inflammatory mediators. LPS, derived from enterobacteria, binds to toll-like receptor (TLR) 4, which triggers a series of signaling pathways leading to NF-κB or AP-1 activation and inflammatory mediator gene expression [13]. Various methods have been used to block these signaling pathways. However, clinical trials with anti-cytokine agents, such as anti-TNF-α antibodies and soluble IL-1 receptor antagonists, have proven ineffective in the treatment of sepsis [14, 15]. These findings suggest that sepsis is not only a disease of a single inflammatory cytokine but that it involves complex pathways involving various cytokines, suggesting that blocking only one cytokine may not be curative because of the orchestrated cross-talk among inflammatory mediators. Meanwhile, it has been shown that LPS stimulation activates NF-κB, leading to target gene transcription [16]. Various methods have been used to suppress NF-κB. However, these methods have been limited in their clinical use because of their low specificity [17, 18].

C-Fos/AP-1 directly controls the expression of inflammatory cytokines by binding to AP-1 motifs in the promoters of these genes [19]. T-5224 was designed and synthesized to be a selective inhibitor of c-Fos/AP-1 using 3D pharmacophore modeling based on a crystal structure of the AP-1-DNA complex [9] and found that selective inhibition of c-Fos/AP-1 resolves arthritis [10]. We have previously shown that T-5224 inhibits serum proinflammatory cytokines, such as TNF-α, IL-1β, and IL-6, in non-lethal LPS-induced AKI in mice (LPS 6 mg/kg body weight) [11]. However, we did not evaluate survival. In the present study, we demonstrated that T-5224 inhibited serum TNF-α expression and improved survival in lethal LPS-induced AKI (LPS 10 mg/kg body weight). HMGB-1 is classically known as an intracellular DNA-binding protein, released by endotoxin-stimulated activated macrophages after 12–18 h. HMGB-1 has been implicated as a late mediator of lethal systemic inflammation in an LPS-induced septic model [5]. Ulloa et al. previously reported that ethyl pyruvate prevented the release of early (TNF-α) and late (HMGB-1) inflammatory mediators through the transcription factor nuclear factor-kappa B (NF-κB) signal transduction pathway [6]. Our study shows that lethal LPS-induced AKI resulted in higher serum HMGB-1 concentrations and that T-5224 inhibits serum HMGB-1 concentration, leading to reduced mortality in mice with LPS-induced AKI. This finding is consistent with previous studies [5, 6]. Based on our study and previous studies, HMGB-1 is thought to be a more effective therapeutic target for the treatment of lethal LPS-induced AKI as well as other proinflammatory cytokines. However, T-5224 did not completely inhibit serum TNF-α expression in our study. One potential explanation for this could be that genes encoding inflammatory cytokines are transcriptionally activated through NF-κB and AP-1 activation, and that T-5224 selectively inhibits the DNA-binding activity of c-Fos/c-Jun, without affecting binding of other transcription factors such as NF-κB.

In sepsis patients, immunosuppression occurs in addition to hyperinflammation and can lead to lethal infection [20]. Regulation of immunosuppression could also be more beneficial than suppression of hyperinflammation. T-5224 treatment immediately after LPS injection increased serum IL-10 concentrations. IL-10 is an anti-inflammatory cytokine that decreases production of several inflammatory cytokines, such as TNF-α, IL-1β, and IL-6, preventing subsequent death [21]. The balance between inflammation and immune response following T-5224 treatment might affect the survival rate. Furthermore, T-5224 may have potential benefits in patients with the immunosuppression state. However, further studies are needed to evaluate this possibility.

The serum BUN and creatinine concentrations were used in this study as indicators of renal function in LPS-induced AKI. The increase of serum creatinine level and oliguria during sepsis often appears after the window of opportunity for effective therapy. Because septic AKI is characterized by a distinct pathophysiology, new biomarker for early detection of AKI is needed [22]. A recent study demonstrated that septic AKI patients have higher detectable plasma neutrophil gelatinase-associated lipocalin compared with non-septic patients [23]. The utility of such biomarkers may be of particular importance in lethal septic patients, because early detection of AKI would significantly decrease morbidity and mortality related to AKI [24].

Sepsis is characterized by hyperactivation of inflammatory cascade and causes organ dysfunction in vital organs such as the lung, liver, and kidney. C-Fos/AP-1 controls the expression of inflammatory cytokines including TNF-α by binding directly to AP-1 motifs in the promoters of these genes [19]. T-5224 is a new drug that selectively inhibits c-Fos/AP-1 binding to DNA. In addition to the effects of T-5224 on LPS-induced AKI [11], we demonstrated that T-5224 attenuated LPS-induced liver injury in mice [25]. T-5224 may beneficial effects in sepsis-induced organ dysfunctions. Further experimental studies will be needed to better understand the potential effects of T-5224 on other sepsis-induced organ dysfunctions such as sepsis-associated acute respiratory distress syndrome.

There were three limitations in our study. First, we did not investigate whether T-5224 specifically binds c-Fos/AP-1 in renal cells. Additional studies will be required to elucidate whether T-5524 inhibits AP-1 activation in LPS-stimulated renal cells. The second limitation was oral administration and the timing and of T-5524. We orally administered T-5224 after LPS injection. We did not measure the plasma concentration of T-5224 after a single oral administration of T-5224 in this study. Aikawa et al. demonstrated that the maximum plasma concentration of T-5224 was 240 ng/ml after a single oral administration of T-5224 (10 mg/kg body weight) in collagen-induced arthritis in mice. T-5224 at 1.5 h after a single oral administration maintained high concentration [10]. Therefore, we assumed that the plasma concentration of T-5224 (300 mg/kg) at 1.5 h after LPS injection was effective enough. Further studies are needed to evaluate this assumption. In addition, sepsis treatments are started several hours after onset in clinical setting. Because most patients with severe sepsis could not take a meal, intravenous administration of T-5524 to treat persistent inflammation is needed to be considered. This limitation is also issued to address in future studies. The last limitation was the histological evaluation. We did not evaluate quantitatively histopathological differences among groups, because sample number is small. Further studies are needed to confirm our data, based on morphology.

## Conclusions

This study demonstrated that a single dose of T-5224 improved the survival by inhibiting expression of a proinflammatory cytokine (TNF-α) and a late mediator (HMGB-1) in lethal LPS-induced AKI. The results of the present study provide new fundamental insights, suggesting that T-5224 may be a novel therapeutic agent for treating sepsis-induced AKI.

## Abbreviations

3D: three-dimensional
AKI: acute kidney injury
AP-1: activator protein-1
bZIP: basic region-leucine zipper
ELISA: enzyme-linked immunosorbent assay
H & E: hematoxylin and eosin
HMGB-1: high mobility group box-1; HMGB-1
i.p.: intraperitoneal
ICU: intensive care unit
IL: interleukin
LPS: lipopolysaccharide
NF-κB: nuclear factor-κB
PVP: polyvinylpyrrolidone
TLR: toll-like receptor
TNF-α: tumor necrosis factor
